# Pre-tertiary education, ethnicity, and attitudes of Asian medical undergraduates towards communication skills

**DOI:** 10.5116/ijme.5c30.988d

**Published:** 2019-01-25

**Authors:** Andrew LS Foong, Chew-Fei Sow, Shamala Ramasamy, Poh-Sin Yap

**Affiliations:** 1 College of Health & Medicine, University of Tasmania, Australia; 2 International Medical University, Kuala Lumpur, Malaysia

**Keywords:** Communication skills, attitudes, ethnicity, culture, school education system

## Abstract

**Objectives:**

This study was aimed at determining whether the pre-tertiary education system and ethnicity have any association with the attitudes of medical undergraduates towards communication skills. It also sought to determine if attitudes should have any relationship with communication skills assessment outcomes.

**Methods:**

A cross-section survey design was performed with 323 participants from two cohorts of medical undergraduates, i.e., first-year (n = 153) and second-year students (n = 170) who completed the Communication Skills Attitude Scale. Participants comprised of the main ethnic groups in Malaysia, i.e., Malays, Chinese and Indians, from different language medium pre-tertiary education backgrounds. Attitude measurements were compared with OSCE outcomes.

**Results:**

There was a significant difference in Negative Attitude Scale between pre-tertiary education system with attitudes towards communication skills (F _(3, 319)_ = 7.79, p = .001), but no significant difference with Positive Attitude Scale (F _(3, 319) _= 0.43, p = .649). There was no significant difference between ethnicity and attitudes towards communication skills with PAS (F _(2, 320)_ = 0.66, p = .519) and NAS (F _(2, 320) _= 1.24, p = .291). Students from Chinese medium education system had stronger negative attitudes with a mean score of 14.7 (n = 56, SD = 3.6) for primary school levels and 15.9 (n = 17, SD = 3.0) for secondary school levels, compared with others. There was no significant prediction of student’s attitudes towards assessments outcomes.

**Conclusions:**

Preliminary findings from the small data pool suggest indicative relationships requiring further studies with more participants and proportionate pre-tertiary education system backgrounds.

## Introduction

Cultural diversities do present challenges for clear communication in various facets of societal functioning.^1^ Being socially transmitted, culture permeates into the mindset of people of different cultures influencing signs and symbols for a different meaning of words, gestures, interpretations, and reactions.^2^

The subject of doctor-patient communication has been well documented including the challenges of East-West cultural differences whereby doctors in the East tend to be perceived in a hierarchy, as figures of authority that may not be questioned.^3^^,^^4^ One study in Indonesia reported on the preference for a participatory approach, but patients were seen as being unprepared for a participatory communication style, not helped by doctors’ lack of communication skills.^5^ Patients are generally dissatisfied with doctors’ communications with them.^6^ In a similar vein, Asian students are perceived to lack assertiveness especially from the ethnocentric prism of the predominant western perspective. They are moulded (encultured) by authoritarian teachers to be obedient students, as passive receivers of didactic teaching, and examination driven rote learners.^7^ The impact is on competent clinical functioning as acknowledged by Makoul and Schofield^8^ when they outlined recommendations for teaching and assessment of communication in medical education. The study of medicine tends to be viewed within the realm of hard physical sciences, all competent physicians should have, with little attention being paid to soft behavioural sciences^9^ until relatively recently.^8^^,^^10^ Emphases on physical sciences is evident from entry requirements into medical school further reinforcing them as priorities which medical students should focus on which become internalised in their attitudes.

A major soft science challenge is that of communication skills’ short-comings in the broader undergraduate population including medical^11^ and postgraduate students in Malaysia.^12^ This is also a problem leading to medical graduates dropping out during their internships due to poor communication capabilities.^13^ Besides culture-based attitudes, the literature has highlighted linguistic issues^14^ to be the major barrier in students’ learning of communication skills. In the Malaysian context, most students speak 2-3 languages due to the school education system. Studies on communication skills in health care have mostly been conducted in the Western world. This may limit the validity of the evidence for the Asian context which has a strong cultural influence^15^^,^^16^ impacting on perceived interactions^17^ and self-perceived communication capabilities.^18^

Although the official language in Malaysia is Bahasa Malaysia (Malay language) with government department communication in that language, the industrial and commercial sectors, which sustain the economy, consider the ability to speak effectively in English to be key assets for their employees.^19^ Communication problems including difficulty in expressing themselves have been identified due to low proficiency in English language.^20^

Malaysia has a unique school system. Prior to independence in 1957, to meet the needs of the three main ethnic groups; Malays, Chinese and Indians, the British facilitated different types of education systems, inadvertently reinforcing segregation of the racial, social, linguistic and cultural groups.^21^ Attempts have been made to provide one common system of education to foster national integration amongst the different ethnic groups, whilst also allowing for alternative streams of education delivery at both primary and secondary school levels, to satisfy divergent needs and interests.^22^ Basically, the primary and secondary schools are streamed according to the three language mediums of instructions, namely, Malay, Chinese (Mandarin) and Indian (Tamil) languages to cater for the main racial groups. The stipulation is for a compulsory pass in Bahasa Malaysia but not in English Language with consequences from its neglect.^23^ However, in tertiary education, almost all of the instructions are in the English Language. The pre-tertiary education system has been seen to contribute to students’ lack of confidence in communication,^24^ with poor ratings in English proficiency when at tertiary education abroad, such as in Australia.^25^

Each of these ethnic groups has kept their strong ethnic identity in the form of linguistics, social values, beliefs and traditional cultural customs,^26^ which inherently play a part in moulding attitudes, as with the theory of linguistic relativity where language could influence thinking.^27^^,^^28^ Our language capabilities are determined by our vocabulary capacities and such capacities and capabilities could, in turn, influence levels of thinking, behaviours, and values.^29^ Inherent sociocultural values transmitted may be manifested in the form of cognition, beliefs and behaviours, such as from influences by significant others.^30^

Concerning education, the picture is further complicated by an Asian culture which is considered a Confucian Heritage Culture.^31^ Students within such a culture are viewed as passive, reproductive and surface learners.^32^ Such enculturation impacts on teaching and learning,^33^ and learning styles.^15^ Apart from learning style differences between cultural groups, variations exist in the rules for general discourse in verbal communication. Discourse rules govern such aspects as the use of nonverbal behaviours and silence as a communicative device.^34^ Such behavioural manifestations appear to persist regardless of whether students are in Asian or English-speaking countries, which leads to the assertion that they are determined by situation-specific factors of teaching approaches, learning habits, learning requirements, and language proficiency, rather than culture.^31^

Given the above background, one question is whether enculturation through the various education systems and ethnicity could provide insights or explanations for the presenting attitudes held by students towards such functions as communication skills, and ultimately, their performance in clinical settings.

In view of communication skills being of primary importance for professional functioning, further insights on whether the national social policy of school streaming based on language and ethnicity, can shed light on the matter of communication skills challenges amongst medical school undergraduates in Malaysia.

The aims of the study are, firstly, to investigate whether any of the identified pre-tertiary language-based education systems are associated with the attitudes of medical students towards communication skills. Secondly, as language is linked to ethnicity, the study investigates whether ethnicity is associated with the attitudes of medical students towards communication skills. Additionally, the study examined whether attitudes of medical students towards communication skills are predictive of clinical skills assessment outcomes.

### Methods

### Study design

This study draws upon a cross-sectional survey design with data collected from two cohorts of medical students from Year 1 and Year 3 at a private medical school. Cross-sectional design is utilized as the aims of the study are, firstly, to determine the association of pre-tertiary education systems attended with attitudes towards communication skills, and secondly, to investigate the association of ethnicity with attitudes towards communication skills at the present moment. Participants were recruited using a purposive sampling method. The inclusion criteria for this study is all actively enrolled first year and third-year medical students. Medical students from Year 1, were three months into their study and were not exposed to the communications teaching in the curriculum. They were recruited for this study based on the justification that differences in attitudes towards communication skills could be identified following further experiences in the course of medical studies. The possibility for change in attitudes is real, in light of experiences students may encounter over the course of their medical education,^35^ therefore, also serving to shed some light on Tran’s31 assertion of situation-specific factors. Students from Year 3 are those who had been trained for three years in communication skills in a simulated environment along with some exposure in the clinical environment with real patients. They were selected as they had engaged in the clinical skill assessment, thus, enabling an investigation on whether attitudes of medical students towards communication skills are predictive of clinical skills assessment outcomes.

### Participants

A total of 388 students were invited to participate in this study in the lecture hall, following attendance at unrelated lectures. Of the total invited, 323 (83.2 %) completed the self-administered questionnaire comprising of demographic information and a measure of their attitudes towards communication skills. The participants comprised of 153 out of 185 students (82.7%) from the first year of the medical programme (Year 1), and 170 out of 193 participants (88.1%) from the third year (Year 3).

Participation of all samples in this study was on a strictly voluntary basis and the rights to withdraw at any time were verbally explained to them and also stated on the consent form. Consent included using the data collected and the clinical skills assessment results for the purpose of data analysis as well as publication. All aspects of ethical clearance were obtained from the University Research and Ethics Committee.

### Instrument

With the aim of the study outlined, the 26-item of Communication Skills Attitude Scale (CSAS) was employed to measure the students’ attitudes towards communication skills.^36^ The reliability coefficient for all CSAS items was 0.873 for subscale 1, and 0.805 for subscale 2.^36^ It is a 5-point Likert-scale ranging from 1 (strongly disagree) to 5 (strongly agree) which has been adopted by Ullah and colleagues (2012),^37^ in their study which demonstrated validity and reliability in the Malaysian context.  The CSAS items comprises of two categories i.e. the positive attitude subscale (PAS) and the negative attitude subscale (NAS) with modest modification.^37^ The PAS scores included items 4, 5, 7, 9, 10, 12, 14, 16, 18, 21, 22, along with revised scoring for items 2, 15, 19, and 26.  The NAS scores included items 1, 3, 6, 8, 11, 17, 24 and revised scoring for item 23.   Both PAS and NAS scores were calculated based on the summation for their items. They ranged from 15 to 75 for PAS, and 8 to 40 for NAS with the higher scores indicating stronger attitude accordingly.

### Procedure

The 323 students who had agreed to participate in the study were provided with explanations of the project in the lecture hall, followed by reinforcement of their rights to withdraw from the study at any time. Data collection was undertaken on two separate occasions for each of the Year 1 and Year 3 cohorts. Informed consent forms were distributed for signature and returned to the researchers. The students retained duplicate copies. The demographic questionnaire and CSAS were distributed for self-completion by the students and collected individually as they completed them, for data entry and analysis.

### Data analysis

The Statistical Analysis System (SAS) was used to analyse the data.  In addition to the descriptive demographic data, t-tests were used to compare the education systems and CSAS scores between Year 1 and Year 3 cohorts, respectively.  Analysis of variance (ANOVA) was conducted to assess the significant differences between education systems and ethnicity variables, respectively, with PAS and NAS scores for each cohort.

An assessment was undertaken to determine if the attitudes held had any association with actual test performance outcomes. To determine that, the Objective Structured Clinical Examination (OSCE) results were extracted from the Year 3 students to provide a pattern for any association between test performance on PAS and NAS scores. The OSCE consisted of 16 stations with a duration of 5 minutes for each station. The OSCE blueprint comprised of 8 history taking stations and eight physical examination stations.  In this case, communication skills were assessed as a major part of the OSCE, mostly, in the history-taking stations. The communication skills weighting was approximately 50% of total marks for each of the history-taking stations and about 10% of the total marks for each physical examination station. The OSCE scores were not available for Year 1, as they had not undertaken the OSCE at the time of the study. Essentially, through logistics regression, attitude measurements were compared with actual communication skills assessments outcomes to determine if those who had failed in the assessments were also those with negative attitudes towards communication skills.

## Results

Descriptive demographics of the two cohorts are presented in Table 1. The majority of the participants were female (n = 188, 58.2%), and of Chinese ethnicity (n=191, 59.1%). The majority of Year 1 participants were from an English education system, at both primary (n=71, 46.4%) and secondary levels (n=105, 68.6%). On the other hand, most of Year 3 participants were from a Chinese education system at primary level (n=71, 41.8%).  On average, Year 1 participants, had higher PAS mean scores of 64.0 (n=153, SD=6.8, p = .002), out of a maximum score of 75, and lower NAS mean scores of 15.1 (n=153, SD=3.9, p = .001), out of a maximum of 40, compared to Year 3 PAS mean scores of 61.7 (n=170, SD = 6.4, p = .002) and NAS mean scores of 16.7 (n=170, SD = 4.2, p = .001).

**Table 1:** Demographics of students in Year 1 and Year 3

Characteristics	Year 1 n (%)	Year 3 n (%)	p-value
Gender			
Female	95 (62.1)	93 (54.7)	0.179
Male	58 (37.9)	77 (45.3)	
Ethnicity			
Malay	16 (10.5)	27 (15.9)	0.186
Chinese	87 (56.9)	104 (61.2)	
Indian	30 (19.6)	23 (13.5)	
Others	20 (13.1)	16 (9.4)	
Language medium at Primary School			
Malay	15 (9.8)	56 (32.9)	0.001
Mandarin/Chinese	56 (36.6)	71 (41.8)	
Tamil	6 (3.9)	4 (2.4)	
English	71 (46.4)	37 (21.8)	
Others	5 (3.3)	2 (1.2)	
Language medium at Secondary School^a^			
Malay	29 (19.0)	70 (41.2)	0.001
Mandarin/Chinese	17 (11.1)	35 (20.6)	
English	105 (68.6)	63 (37.1)	
Others	2 (1.3)	2 (1.2)	
Communication Skills Attitude Scale (CSAS)	Mean (± SD)	Mean (± SD)	
Positive Attitude Subscale (PAS)	64.0 (6.8)	61.7 (6.4)	0.002
Negative Attitude Subscale (NAS)	15.1 (3.9)	16.7 (4.2)	0.001

NOTE: 7 and 32 missing student information for semester 1 and 3 respectively Chi-squared statistic were performed on categorical variables, fisher exact statistic performed on those with expected cell counts p < 5, and t-statics were used for continuous variables, p < 0.05, statistically significant a No Tamil medium school in Malaysian secondary school system

In relation to the first aim of the study on whether there are any significant differences between the pre-tertiary education system with attitudes towards communication skills, no significant results were found with PAS (F_ (3, 319) _= 0.43, p = .649), and NAS (F _(3, 319)_ = 7.79, p = .001). For the second aim of the study there was no significant difference between ethnicity and attitudes towards communication skills with PAS (F _(2, 320)_ = 0.66, p = .519) and NAS (F _(2, 320) _= 1.24, p = .291). In all cases, those from Chinese medium education system backgrounds had stronger negative attitudes with a mean score of 14.7, (n = 56, SD = 3.6) for primary school levels and 15.9 (n = 17, SD = 3.0) for secondary school levels, compared with others (see Table 2).

Comparatively, for Year 3, there was an observation of greater NAS (see Table 3) for the Mandarin education system, with a mean score of 15.8 (n = 71, SD = 4.2) for the primary school level, and a mean score of 15.9 (n = 35, SD = 3.9) for the secondary school level. Those from the English education system had the lowest NAS, with mean scores of 14.2 (n = 37, SD = 3.6) for the primary school group, and 14.6 (n = 63, SD = 3.7) for the secondary level school group. No comparisons were made for the Tamil education system as they do not exist at secondary education level.

**Table 2:** Differences between demographics and communication skills attitude scale (CSAS) in Year 1 students

Characteristics	Positive attitude subscale (PAS)		Negative attitude subscale (NAS)	
	Mean (± SD)	F (p-value)	Mean (± SD)	F (p-value)
Age in Years				
18 -20	64.0 (6.5)		13.7 (3.4)	
20 - 24	63.1 (9.0)		12.4 (4.1)	
25 and above	68.0 (4.7)	0.88 (0.418)	9.8 (4.2)	3.49 (0.033)
Gender				
Female	63.9 (6.9)		13.7 (3.5)	
Male	64.1 (6.7)	0.01 (0.905)	13.0 (3.6)	1.26 (0.263)
Ethnicity				
Malay	63.1 (9.8)		13.6 (3.3)	
Chinese	63.6 (7.0)		13.8 (3.7)	
Indian/Other	64.8 (5.2)	0.66 (0.519)	12.8 (3.4)	1.24 (0.291)
Language medium at Primary School				
Malay	64.9 (4.8)		14.0 (2.3)	
Mandarin/Chinese	64.0 (6.9)		14.7 (3.6)	
English	63.6 (7.4)		12.4 (3.7)	
Tamil	64.7 (4.3)	0.19 (0.900)	13.5 (2.5)	4.67 (0.004)
Language medium at Secondary School^a^				
Malay	64.9 (3.9)		14.2 (3.0)	
Mandarin	64.5 (5.2)		15.9 (3.0)	
English	63.6 (7.6)	0.43 (0.649)	12.8 (3.6)	7.79 (0.001)

ANOVA statistic, p < 0.05, statistically significant a No Tamil medium school in Malaysia secondary school

With regard to the third aim of the study, to determine if there should be any predictions of student’s attitudes towards communication skills and clinical skills assessments outcomes, no significant results were found. However, a closer examination of the data set suggests possibilities for relationships which may well be more transparent with a higher number of participants that were available for this study.

The tracking of students in the Year 3 cohort who undertook the OSCE indicated that those who failed this examination were also those who had presented with negative attitudes towards communication skills. Proportionally, the failure rate percentage for those from a Mandarin education were relatively higher than others. Those from the Chinese primary school (and secondary school) group had a failure rate of 7.35% (11.1%) compared with 3.85% (1.54%) for the Malay education, and 2.78% (4.92%) for the English education system. No failures were recorded for the Indian education system group. Although the results suggest those from the Chinese education system were most negative towards communication skills, it should be noted that those who failed were not all from that group. Within the ethnic groups,the failure rate for Malays was 8%, compared with Chinese at 5.1%, and Indians at 2.6%.  Logistics regression was used to predict OSCE outcomes about the CSAS scores. The odds were that Year 3 students were 18% more likely to fail their OSCE, for every one unit increment in their negative attitude toward communication skills.

Regarding examination success, the findings suggested a higher tendency for those with higher NAS to fail the OSCE’s, i.e., those from the Chinese/Mandarin education system and the Chinese ethnic group.

**Table 3:** Differences between demographics and communication skills attitude scale (CSAS) in Year 3 students

Characteristics	Positive attitude subscale (PAS)		Negative attitude subscale (NAS)	
	Mean (± SD)	F (p-value)	Mean (± SD)	F (p-value)
Age in Years				
18 -20	59.8 (4.4)		16.1 (3.6)	
20-24	62.1 (6.1)		15.0 (4.0)	
25 and above	57.7 (14.2)	2.24 (0.110)	11.8 (3.1)	2.63 (0.075)
Gender				
Female	62.2 (6.0)		14.5 (3.6)	
Male	61.0 (6.9)	1.46 (0.228)	15.5 (4.3)	2.97 (0.087)
Ethnicity				
Malay	63.4 (4.6)		15.9 (4.3)	
Chinese	60.3 (7.0)		15.5 (4.0)	
Indian/Other	64.4 (4.6)	7.48 (0.001)	12.9 (2.8)	7.42 (0.001)
Language medium at Primary School				
Malay	63.1 (4.8)		14.4 (3.9)	
Mandarin/Chinese	60.0 (7.8)		15.8 (4.2)	
English	62.6 (5.3)		14.2 (3.6)	
Tamil	62.2 (5.9)	2.85 (0.039)	14.5 (2.9)	2.01 (0.114)
Language medium at Secondary School^a^				
Malay	63.0 (4.9)		14.8 (4.2)	
Mandarin	58.8 (9.1)		15.9 (3.9)	
English	61.9 (5.6)	5.71 (0.004)	14.6 (3.7)	1.35 (0.263)

ANOVA statistic, p less than 0.05, statistically significant a No Tamil medium school in Malaysia secondary school

## Discussion

The majority of the students being female is consistent with the global picture,^38^^-^^40^ but figures in the USA appear to indicate falling female students since its peak of 51% in 2003.^41^  The majority of students being of Chinese ethnicity is not unusual in a privately funded university in Malaysia, due to a political system that imposes quotas based on race for admission into medical secondary education systems in the public universities.^42^^-^^44^  Hence, many who are not of the Malay ethnic groups who aspire towards a career in medicine, are left with little option but to pursue medical studies at privately funded universities, if they can afford them.

Whilst positive towards communication skills as a whole, on further analysis, the findings suggest different attitudes prevailing between the various sub-groups. In particular, those from the Chinese education background and the Chinese ethnic group are more negative towards communication skills, whilst those from the English education system are more positive. A larger sample size than was available for this study may well have presented a more significant finding in this respect. Such preliminary findings may suggest the role of pre-tertiary education systems and ethnicity in influencing attitudes towards communication skills. It raises the question of contributory factors for such a difference between the various education systems and ethnicity. The issue of ethnicity and its inherent cultural traits is a challenging one including the need for sustained, concerted action from the community if there should be the perceived desire for it. On the other hand, the link with the pre-tertiary education system is a matter that may be more amenable for appropriate action by government policy. It deserves further exploration, given the significant role in both primary and secondary education systems play in moulding the minds of children for the future.

Further examination of the data suggests complex interactions between the variables in this study. One striking aspect is the change in attitudes from Year 1 to Year 3, where it appears that the older Year 3 students have changed attitudes towards the negative for communication skills possibly due to such matters as enculturation exposure which may exist as they progress through their medical education. However, it is inconclusive due to the cross-sectional sampling, and a longitudinal study is needed for verification. Rees and Sheard^45^ found that as age increased, PAS scores decreased. Changes of a similar nature have also been highlighted in a systematic review of 11 studies on medical students and 7 on residents, with specific regard to empathy, a core aspect of communication skills.^35^ Longitudinal data suggested a decline in empathy during medical school education and residency, for a range of reasons including the presence of vulnerabilities to values of idealism, enthusiasm, and humanity at the beginning of medical school.^46^ Those values diminish in time following exposure to clinical realities.^47^

Results from this study, suggest pre-tertiary education, and by virtue of that, the process of enculturation over the years may influence attitudes and behaviours. Asians tend to hold collectivistic values that influence their behavioural manifestations.^16^ Within the collectivist background, there is also the Confucian influence for the Chinese group. Behavioural manifestations from those values may be more pronounced, such as when communicating in the presence of elders, or being passive, reticent, and over-dependent on the teacher, often following without questioning.^48^ As a collectivist society, Asian culture is such that modesty and social harmony is highly regarded to the extent that they tend to be reserved about judging themselves positively^16^ and not challenging the status quo, as manifested by their unwillingness to ask questions or speak up in class.^48^^-^^50^ Although all three main racial groups in the study are considered collectivistic, it seems that those from the Chinese group appear to be more impacted by their closer Confucian heritage.

As a matter of perspective, it should be noted that the phenomenon of unwillingness to ask questions or speak up in class, in both Asian and English-speaking countries, has also been attributed to situation-specific factors of language proficiency,^51^ teaching methodologies, learning requirements, and learning habits, rather than cultural factors.^31^

In this study students who failed the OSCE’s were all identified as being from the list of those who held negative attitudes towards communication skills. Such a finding is consistent with the theory that attitudes influence behaviours^52^ with impacts on learning outcomes.^53^ Paradoxically, taking the study population as a whole, the results indicate the Chinese to be 40% less likely to fail the OSCEs compared with the Malays.

One question is whether those from a Chinese education background are better at test performance. What has been established is that competition and achievement pressure is high in Asian classrooms^54^ and appears to be more prominent amongst the Chinese education system. Great efforts are expended towards achieving high grades. One possible motivator is the Chinese students’ need to achieve higher than their Malay counterparts who are given preference for public university admission but with lower grades than them. In many cases the higher grades may be at the expense of meaningful learning as methods for achieving these may be based on dogmatic memorisation rather than understanding and being able to apply appropriately in context.^55^

A further question is whether the OSCE, based on the Westernised model and philosophy, is a reliable test for measuring communication skills in the Malaysian context. The issue of reliability varies from personal biases to the high anxiety testing method.^56^^-^^58^ Such issues are far more complex than this study can extrapolate. For instance, the long-held narrative that Asian students are reticent and passive learners,^59^^-^^60^ is not helpful as research evidence suggest that many Asian students do have a strong desire to engage in classroom activities.^61^ The cultural background could be a factor affecting student behaviour, reaction, and interactions in the learning environment. Students from the Confucian Heritage Culture may hold a different perspective on what they consider appropriate behaviour in the classroom environment.^32^ It is a complex matter often simplistically explained by reasons including shyness for such behaviour.^62^ Communication skills learning are also determined by other factors including teachers, clinical supervisors, psychodynamics that breaks down barriers and organisational climate.^63^ They include the teachers’ skills at teaching communication skills, incorporating modeling of their own passions for its significance to garner students’ interest, curiosity and motivation towards wanting to develop and aspire to be competent practitioners.

More importantly, consideration is needed for the inherently strong impact of culture, particularly concerning the Confucian heritage where learners are more reflective than impulsive,^64^ tending to prefer a slower and measured response compared with the spontaneity and instantaneity expectations of the Western approach.^65^ Unless explicit, consistent training over what may be considered an adequate period for such cognitive development and functioning to occur,^66^ together with an articulation of required expectations, the reliability of the existing OSCE structure may be questionable within the Asian education context.

The above discussions lead us to the question of whether our study’s findings are comparable with other studies of other ethnic groups such as those from the Western world. It is pertinent given the increasing mobility of students from various parts of the world to tertiary institutions in the Western world. Proportionately, PAS and NAS amongst the participants in this study were comparable to participants from the UK, such as in Leicester and Nottingham where it was suggested the findings of higher NAS scores was due to non-white students at Leicester (which happens to have more non-white students) who have different cultural beliefs about the importance of communication skills within medical practice.^45^

One of the problems with self-administered attitudinal measures, especially with cross-cultural differences, is with discrepancies in describing and interpreting attitude not directly observable. It can be influenced by subjective experiences, in contrast with actual thoughts and feelings affected by such variables as self-image and social acceptance.^67^^-^^68^ For instance, in investigating racial differences in doctors’ information-giving and patients’ participation, racially discordant interactions, and communication patterns perpetuated patient passivity.^69^ Poorer physician-patient communication led to lower trust further compounding the caring relationship.^70^ As measurement tools are mostly self-assessments in nature,^71^ differences may be manifested due to questionnaire response styles,^72^ including participants unwillingness to reveal their true attitudes where they may be seen to be socially unacceptable.

### Limitations

The study design, being cross-sectional, was only able to capture data from medical students at one point in time. It does not provide an accurate picture of changes and differences between the two cohorts. Notwithstanding that, data comparisons suggest emerging differences between Year 1 and Year 3 cohorts with their attitudes. It requires further exploration by a longitudinal follow-up with the Year 1 cohort to determine if exposure over time could bring about changes in attitudes.

Given the disadvantages of self-administered attitude measures discussed earlier, and in this case, being self-administered in a group in a lecture hall, there could be issues such as lack of conscientious responses.  The study with a greater number of students from the Chinese ethnic group could potentially affect the generalisability of the results to the entire medical student population in Malaysia, where the main racial groups of Malays make up 51% of the population, Chinese 24.2%, and Indians 7.2%.^73^ However, it remains representative when it comes to the private medical education sector given the race-based quota system indicated at the beginning of the discussion.

## Conclusion

The findings suggest some association between pre-tertiary education and ethnicity with medical students’ attitudes towards communication skills learning in Malaysia. There are indicative relationships which deserve further studies including with higher numbers of participants and proportionate pre-tertiary education system backgrounds. The preliminary findings serve to reinforce the need to determine the reasons for negative attitudes towards communication skills learning. In this respect, a qualitative study would help identify the reasons for poor communication skills amongst medical students in Malaysia.

### Conflict of Interest

The authors declare that they have no conflict of interest.
